# Machine Learning for Sensorless Temperature Estimation of a BLDC Motor

**DOI:** 10.3390/s21144655

**Published:** 2021-07-07

**Authors:** Dariusz Czerwinski, Jakub Gęca, Krzysztof Kolano

**Affiliations:** 1 Department of Computer Science, Lublin University of Technology, 20-618 Lublin, Poland; d.czerwinski@pollub.pl; 2 Doctoral School, Lublin University of Technology, 20-618 Lublin, Poland; 3 Department of Electrical Drives and Machines, Lublin University of Technology, 20-618 Lublin, Poland; k.kolano@pollub.pl

**Keywords:** temperature estimation, machine learning, BLDC, electric machine protection

## Abstract

In this article, the authors propose two models for BLDC motor winding temperature estimation using machine learning methods. For the purposes of the research, measurements were made for over 160 h of motor operation, and then, they were preprocessed. The algorithms of linear regression, ElasticNet, stochastic gradient descent regressor, support vector machines, decision trees, and AdaBoost were used for predictive modeling. The ability of the models to generalize was achieved by hyperparameter tuning with the use of cross-validation. The conducted research led to promising results of the winding temperature estimation accuracy. In the case of sensorless temperature prediction (model 1), the mean absolute percentage error MAPE was below 4.5% and the coefficient of determination R^2^ was above 0.909. In addition, the extension of the model with the temperature measurement on the casing (model 2) allowed reducing the error value to about 1% and increasing R^2^ to 0.990. The results obtained for the first proposed model show that the overheating protection of the motor can be ensured without direct temperature measurement. In addition, the introduction of a simple casing temperature measurement system allows for an estimation with accuracy suitable for compensating the motor output torque changes related to temperature.

## 1. Introduction

Permanent magnet electric motors, in particular permanent magnet synchronous (PMSM) and brushless direct current (BLDC) motors, have gained popularity over the past decade. It is visible both in industrial solutions such as servo drives and actuators, but also in household appliances and traction applications. This is due, inter alia, to the fact that the use of permanent magnets allows the miniaturization of devices (due to the much higher power density in motors), which also increases their reliability and energy efficiency. The elimination of one of the most susceptible to damage elements of DC electric motors, i.e., the mechanical commutator and brushes, made it possible to use BLDC motors in applications requiring increased durability. The authors in [1] presented the percentage share of individual failures in induction motors, which shows that more than 40% of failures are caused by bearing failures, while 38% are problems with the machine stator. Therefore, it can be concluded that in DC brushless motors, damage to these elements will also have a significant impact on the reliability of the device. Diagnostics and detection of BLDC motor failures are devoted to many studies, including methods consisting of monitoring the motor current waveform [2,3,4], as well as using built-in Hall sensors [5] or additional vibration measurement [6]. A detailed description of faults occurring in brushless motors and the classification of diagnostic methods can be found in [7]. The authors point out that the detection of damage is also possible by measuring the motor temperature.

The analysis of thermal phenomena in electric motors is important for their proper functioning and the possibility of preventing and detecting faults. Excessive temperature rise can destroy the insulation of the stator winding and lead to a short circuit. The increased operating temperature of the motor causes both the aging of the bearings and the degradation of the rotor permanent magnets, which in turn shortens the remaining useful life of the machine. Zhang et al. in [8] also emphasize that the motor life is reduced by 50% for every 10 °C above the maximum temperature limit set by the manufacturer. One of the methods used by manufacturers of electric motors to protect against long-term operation at the upper operating temperature limit is the oversizing of the device or the use of an additional cooling system in the form of a fan placed on the motor shaft. Another cooling method in the case of high-power traction motors is the forced circulation of the coolant in a casing specially designed for this purpose.

According to [9], there are three basic types of thermal losses in permanent magnet motors. The first of these are the losses in the copper winding, the value of which depends on the flowing current. Another is the core iron losses, which mainly depend on the stator voltage. The last type of losses is mechanical, which is influenced by the motor speed. Stator winding insulation is particularly exposed to the effects of temperature and the thermal aging process. Moreover, if the temperature of the permanent magnet motor winding cannot be effectively controlled, the heat will be transferred to the rest of the components through the casing and the air gap, leading to heating of both the bearings and the permanent magnets. The authors in [10] emphasize that the increase in temperature of permanent magnets causes their partial demagnetization, which leads to a drop in motor output torque. If the critical temperature value characterizing a given permanent magnets material is not reached, the process is reversible. Otherwise, the magnets are permanently and irreversibly damaged, resulting in worse motor performance.

The above-mentioned phenomena show that there is a need to monitor the temperature inside DC brushless motors. Therefore, some BLDC motor manufacturers decided to install factory-built winding temperature sensors. This solution entails an increase in the production costs of the device and constitute another element of the machine that may be damaged. On the other hand, the installation of the temperature sensor by the user requires a lot of time and effort, as well as knowledge about the design of the device itself. 

In order to optimize costs and eliminate the need for sensors, scientists have made a number of attempts to estimate the temperature of individual internal components of electric motors, such as stator winding [11], rotor [12], or bearings [13]. These efforts are mainly aimed at protecting these components from excessive temperature rise. However, studies show that it is also possible to compensate the torque pulsations on the machine shaft caused by the influence of temperature on the winding resistance and demagnetization of permanent magnets [14,15,16,17]. The authors in [18] propose using the BLDC motor thermal model to optimize the trajectory of the industrial robot movements, taking into account thermal constraints. Moreover, based on the research conducted by the authors in [19], it can be concluded that thermal modeling of a traction motor with permanent magnets will have a significant impact on the optimization of V2G (vehicle to grid) systems, because the amount of energy consumed by an electric vehicle is dependent on thermal losses of its motor.

One of the most popular and most effective methods of forecasting the electric motor temperature is lumped parameter thermal networks (LPTN), which simplify the physical model of the motor and allow for temperature estimation based on a set of parameters assigned to network nodes. As described in [20], motor equivalent thermal circuit diagrams can be divided into three basic types depending on the number of nodes in the thermal model. The first is a white box model in which a multi-node network is created that describes the motor based on the theory of heat transfer. In this type of model, there are additional sub-nodes that are designed to even more accurately reflect the actual heat distribution in the machine. Conducting calculations aimed at temperature prediction with such complex network structures requires a lot of computing power and, despite high accuracy, cannot be used for real-time prediction. In addition, creating such accurate models requires knowledge of many parameters and properties of the materials of which the motor is made, as well as expertise in its construction. Therefore, it is obvious that this is difficult to achieve, as manufacturers do not provide complete information about their devices. Light gray box models are another type of thermal equivalent networks. They represent the first degree of simplification compared to the previously described white-box networks and typically have five to fifteen nodes. Thanks to the use of a simpler structure, the complexity of calculations is much lower, although there is still a need for detailed information on the materials and geometry of the motor. In response to the above problems, dark gray box models were created, which have only two to five nodes corresponding to the dominant heat transfer paths and achieve very good prediction accuracy thanks to determining the values of the thermal model parameters based on experimental tests. High efficiency and the possibility of real-time calculations made dark gray box networks popular in the field of thermal modeling of electric motors. However, it is worth noting that they require knowledge of the temperature (application of the sensor) in at least one point in the network, as well as some expertise knowledge of the modeled object. In the literature, a number of publications on temperature estimation based on the created lumped parameter thermal networks of permanent magnet motors can be found [11,21,22].

Another way to predict the temperature of electric motors is to estimate the winding resistance by injecting signals of the appropriate frequency into the stator circuit [12,23,24,25]. Methods of this type allow for real-time temperature estimation and are resistant to changes in motor cooling conditions (damage to the cooling system), because it is assumed that the relationship between winding resistance and temperature is known and does not change with time. However, the introduction of additional signals causes current and voltage distortions, significantly affecting the electromagnetic compatibility of the device. In addition, the injected signals cause torque pulsations that are unacceptable in some applications. 

Thermal modeling of electric motors is also carried out in a purely analytical manner using mathematical [26] and finite element (FEA) methods. It is worth mentioning that there are also hybrid estimation methods such as those described in [27]. An interesting issue concerning motors with permanent magnets is the estimation of the rotor temperature on the basis of the flux measurement with the use of built-in Hall sensors, as described in [5]. However, this method requires the knowledge of the thermal demagnetization constant of the material of which the magnets are made. Moreover, the flux measurement is also affected by the influence of the stator flux, which contributes to erroneous predictions at higher loads.

The modern and very effective methods of estimating the temperature of electric motors include machine learning and deep learning. Their unquestionable advantage is that on the basis of the collected measurement data, a function mapping the relationship between the values of the input features and the output is determined. This means that predictive modeling does not require knowledge of the material properties of a given device or having expertise knowledge about its construction. Both neural networks and other machine learning methods have proven their effectiveness in estimating the temperature of induction motors [28], permanent magnets synchronous motors [9,29,30,31], as well as brushed DC motors [32,33]. Many of the articles on PMSM temperature prediction using machine learning available in the literature use the motor coolant temperature as an input variable of the algorithm [9,29,30,31]. Moreover, the authors in [34] emphasize that the stator temperature is strongly correlated with the exponentially weighted moving average of the PMSM motor coolant temperature, and removing this variable from the feature vector results in a significant decrease in the effectiveness of the prediction algorithm.

In this article, we present a comparison of the effectiveness of selected machine learning algorithms in predicting the temperature of BLDC stator winding for a variable load profile and various cooling conditions. An interesting result was achieved even without the knowledge of casing temperature. In addition, the results of the estimation using information about the temperature on the casing and in the absence of additional sensors were compared. The article is organized as follows: Section 2 describes measurement of the BLDC motor winding temperature and the methods used for data preprocessing; Section 3 describes the machine learning algorithms that were used to develop the models; cross-validation and parameter optimization are described in Section 4; Section 5 and Section 6 present the results of predictive analysis, while Section 7 presents the summary and conclusions.

## 2. Measurements and Data Preprocessing

**Figure 1:**
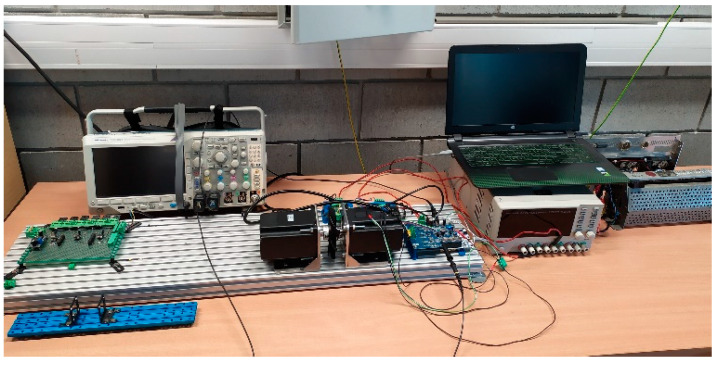
Test bench.

**Figure 2:**
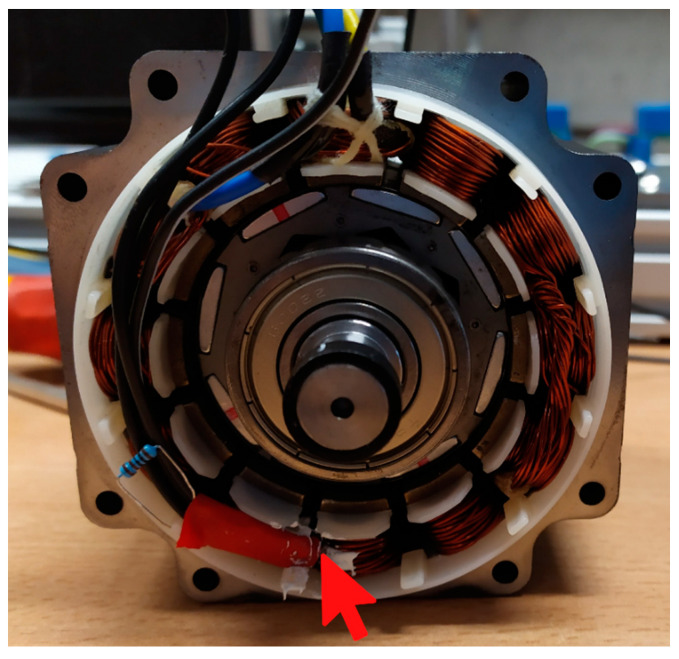
Winding temperature sensor placement (**red arrow**).

**Figure 3:**
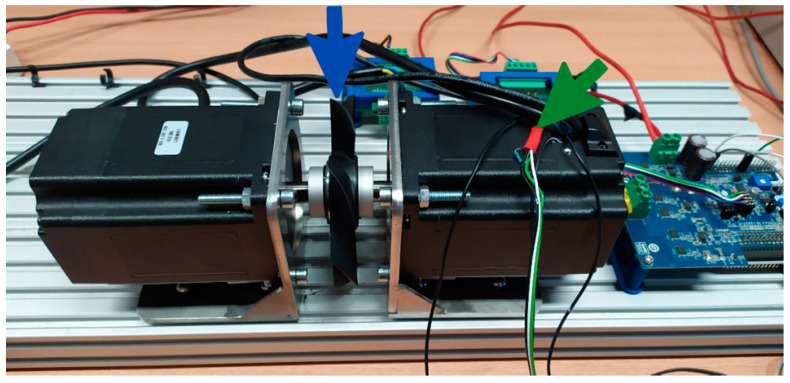
Placement of the cooling fan (**blue arrow**) and the casing temperature sensor (**green arrow**).

One of the key aspects of predictive modeling with the use of machine learning algorithms is the collection of a sufficiently large set of data, which will be split and then used for training, testing, and validating the results. For this purpose, two mechanically coupled brushless DC machines of the same type (Table 1) were installed on the test stand (Figure 1). One of them worked as a motor during the measurements, while the other one worked as a generator and constituted the adjustable load. The BLDC motor was equipped with two analog LM35DZ temperature sensors supported by the Texas Instruments (Dallas, TX, USA), one of which was mounted to the winding ends with a thermally conductive adhesive, as shown in Figure 2, while the other was placed on the motor casing—Figure 3. The control of the tested motor was carried out with the use of the algorithm described in [35,36] to eliminate the possible impact of Hall sensors’ misalignment on the effectiveness of the estimation. The IHM08M1 system, dedicated to work with STM32 series microprocessors supported by the STMicroelectronics N.V. (Amsterdam, The Netherland), was used as the power electronic converter. This converter is equipped with a current measurement system. In addition, the rotational speed of the motor was measured using a preinstalled Hall sensors’ system. Data acquisition was carried out using the STM Studio software dedicated to real-time visualization and data acquisition, but due to the fact that thermal processes are slowly changing, it was decided to sample the measurements at a frequency of 4 Hz.

Over 160 h of BLDC motor temperature measurements were carried out on the test stand for a variable load profile and rotational speed, as well as for various cooling conditions. During the first series of measurements lasting more than 80 h, the motor was tested without any additional cooling system. In the second one, a cooling fan was mounted on the motor shaft (Figure 3). In addition, an air duct has been provided to ensure an adequate flow of cooling air.

**Figure 4:**
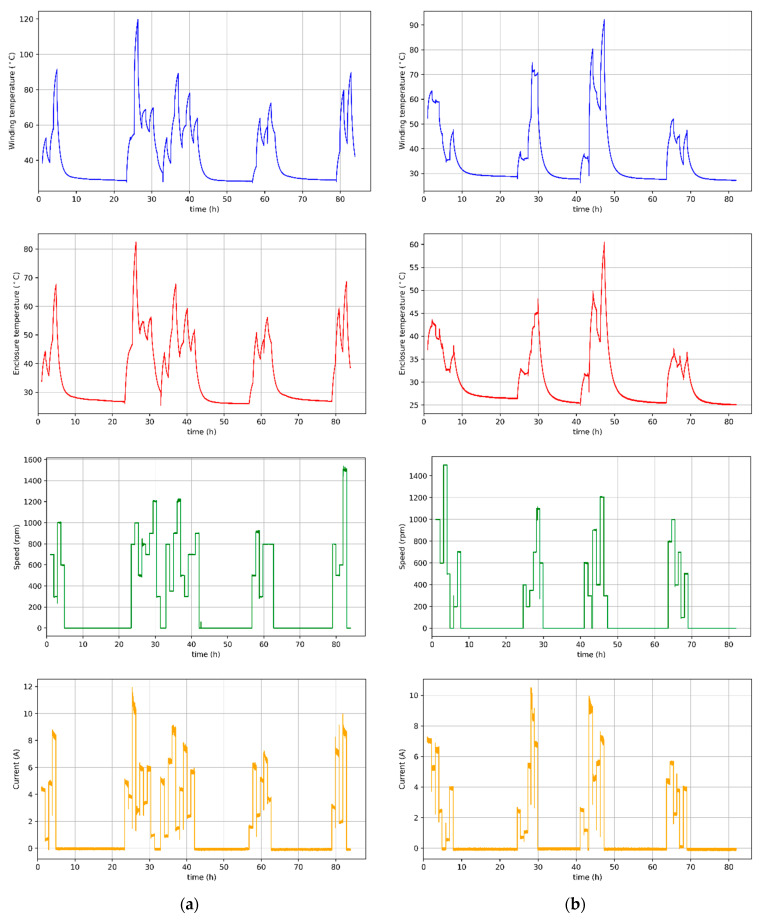
Measurement results of winding temperature (blue), casing temperature (red), speed (green), and current (orange) of motor: (**a**) without cooling; (**b**) with cooling fan.

The results of the measurements are presented in Figure 4a,b. The load was realized by the machine of the same type coupled on the common shaft. Windings of this auxiliary machine were connected to the variable resistance load. Changes of the rotational speed were achieved by changing the target speed of a closed-loop speed controller. It can be seen that after simultaneous load and rotational speed change, the heating or cooling of the motor is visible for a certain time interval. Therefore, it can be concluded that the important information from the algorithm’s point of view will be the time that has elapsed since the last load change. Accurate information about the moment of load change is not available, and its detection is difficult. Therefore, we decided to introduce new features that will constitute a short-term history of the device from previous $N_{h}=14,400$ data records, which corresponds to one hour of measurements (this time was selected on the basis of observations of the obtained waveforms). For this purpose, the mean and standard deviation of the current and rotational speed from the last $N_{h}$ measurements were calculated for each data record, thus creating additional feature variables. Selection of the optimal length of the short-term history of the device, also known as the so-called look-back parameter, is out of the scope of this work and may form the basis for further research in this field. It is worth emphasizing that for results from Figure 4b, a cooling fan was placed on the common shaft with the motor, so it constitutes the additional load. Moreover, temperature is more dependent on the speed than in the case without a fan. Therefore, when the speed is low, the cooling conditions greatly worsen. If the load is high but the speed is also high, cooling is ensured, and the temperature does not rise too much. As an additional input variable, the winding power losses were also introduced, which were calculated from the formula:

(1) $$P_{wl}=I^{2}R_{w}$$

where I is the winding current and $R_{w}$ is the stator winding resistance.

An important issue related to the data preparation is the appropriate division into a training set and a test set. In the case of PMSM motor temperature prediction presented by the authors in [34], the 55 h of measurements were enough to train the algorithm properly. Referring to these studies, the dataset was divided into training and test subsets in the proportion of 70:30. As a result, training sets of more than 50 h were obtained for both experiments. It is also worth adding that in the analyzed case, the division had to be carried out with respect to the record occurrence and not in a random manner. As mentioned before, the temperature inside the BLDC motor depends on the time that has elapsed since the last load and/or speed change, which can be seen as the change in the supplying current. Due to the fact that the data are in the form of a timeseries, it is unacceptable to shuffle them.

Measurement data obtained on the test stand and constituting the basis for training and validation of algorithms are expressed in various units and on a different scale (speed is up to 1500 rpm, and current up to 12 A). The use of data in this form could cause a situation where the speed, due to high values, will dominate the cost function values, making it impossible to obtain any useful information from other features, which would significantly worsen the effectiveness of the algorithms. Moreover, some predictive models may generate larger errors if the individual features are not approximately normally distributed. The exception in this case are decision trees algorithms, which are resistant to different scaling of features. In response to the above problem, in this paper, the feature variables have been standardized as follows. First, the mean μ and standard deviation σ of each feature from the subset of training data were calculated. Then, all samples of a given feature were transformed according to the formula:

(2) $$x_{std}=\frac{x-\mu }{\sigma }.$$

The data transformed in this way have approximately zero mean, standard deviation σ=1, and are appropriately scaled.

## 3. Machine Learning Algorithms

The purpose of machine learning algorithms for regression is to predict the value of the target variable based on the set of independent variables, which are commonly known as features. The algorithm acquires the ability to forecast in the learning process, which consists of providing examples that allow prediction verification. Then, the algorithm modifies its own structure in such a way as to minimize errors. Linear regression models [37] implement the above statements in the form of an equation:

(3) $$\hat{y}=w^{T}x+b$$

where $\hat{y}$ is the output of the algorithm, i.e., the predicted value, $x\in {\mathbb{R}}^{n}$ is the input features vector, and $w\in {\mathbb{R}}^{n}$ is the vector of the model parameters, which are also called weights. This name results from the fact that they allow defining how strong a given feature should influence the output value. At this point, it is also worth adding that it is the weight values that are optimized in the learning process, thanks to which it is possible to improve the effectiveness of prediction. The component b in (3) is called a bias.

An alternative way of introducing bias in the model is to add the component of value 1 to the feature vector. Then, the weight assigned to this component will act as a bias.

The purpose of modifying the parameters of the model is to find a weight vector that will allow correct prediction of the variable. In other words, the weight update procedure should minimize some prediction error function. One of the most frequently used objective functions in regression is the sum of squared errors (SSE):

(4) $$J(w)=\sum_{i=1}^{N}{\left(\hat{y}-y\right)}_{i}^{2}$$

where N is the number of samples. The above formula also has its geometric equivalent as the Euclidean distance between the prediction $\hat{y}$ and the real value y of the target variable:

(5) $$J(w)={\left\|\hat{y}-y\right\|}_{2}^{2}.$$

Since the objective function has been defined as well as the parameter that will be modified, all that remains is to use an optimization algorithm, which can be, for example, the gradient descent. However, note that moving along the gradient decrease may end up reaching a local rather than a global minimum of the cost function. Of course, in many cases, reaching the global minimum is not possible at all, and the solution obtained with this method is sufficient. However, the objective function can have a very complex structure with many local minima, each of which allows for different predictive accuracies. For this reason, scientists have developed many algorithms that are more resistant to getting stuck in small local minima of the cost function and more efficient in terms of computational complexity. Such algorithms include the commonly used optimizer Adam [38].

The main task of machine learning algorithms is to effectively predict specific quantities based on the provided, previously unseen data. It is possible thanks to the previously conducted process of learning the algorithm on the so-called training data. However, from the application point of view, it is most important to generalize well, that is, use the acquired “knowledge” to correctly analyze new data. As previously explained, learning consists in adjusting the parameters of the algorithm based on the determined training error in order to minimize it. The above issue can be treated as an optimization problem, but the test error (also known as the generalization error) should also be as small as possible. Therefore, determining how well a given algorithm will analyze new data is based on the value of the training error and the difference between the training error and the test error. In this way, it is possible to avoid underfitting, which occurs when the algorithm is unable to achieve a sufficiently small value of the training error, and overfitting, the sign of which is a large difference between the training error and the test error. As the algorithm’s complexity (also called capacity) increases, the model’s variance increases, along with the difference between the value of the training error and the test error, and thus the total error of the system. In the opposite situation, the capacity of the algorithm decreases, the bias increases, and the training error (and thus the total error) increases. This means that a very important element in the design of a machine learning model is to establish an appropriate compromise between bias and variance (underfitting and overfitting). This goal is achieved by adjusting the algorithm’s capacity to the true complexity of the problem as well as the amount of training data available. It is a known fact that in practice, there is always some kind of noise and outliers among the real-world data. For this reason, reaching a satisfactory compromise is possible only with the acceptance of training errors resulting from an incorrect classification of outliers. Thanks to this, the prediction of future data will be insensitive to possible noises in the provided test data. In response to the above-presented need to limit or regulate the complexity of the machine learning model, many techniques have been developed that are generally referred to as regularization. Some of them introduce additional constraints on the machine learning model, for example by imposing limits on parameter values. In turn, others extend the objective function with special expressions [39]. Additional restrictions and penalties, if carefully selected, can lead to improved performance on test data. Many methods of regularization are based on limiting the capacity of the model by adding a norm component Ω(w) to the objective function J. This penalty term is used to limit the values in the parameter matrix w. The regularized objective function $\tilde{J}$ can be written as a variable:

(6) $$\tilde{J}(w;x,y)=J(w;x,y)+\alpha \Omega (w)$$

where α∈<0,∞) is a hyperparameter that regulates the strength of regularization with respect to the standard cost function [40]. Expression (6) shows that the minimizing cost function $\tilde{J}$ created in the learning process will result in an optimization of both the original cost function J and some measure of parameter size Ω (or a subset of parameters). A comparison of the regularized objective functions is presented in Table 2.

In this paper, we investigate the effectiveness of temperature prediction by linear regression models as well as those based on decision trees. The following algorithms were tested during the research:

1. Linear regression with the objective function given by Formula (4),
2. Elastic-Net regressor, in which the regularization components are introduced to the objective function (Table 2),
3. Regressor using the stochastic gradient descent optimization algorithm (denoted as SGD),
4. Support vector machine (SVM) with linear kernel,
5. CART (Classification and regression trees) decision trees,
6. AdaBoost—presented in [41], an algorithm that uses boosting to determine the final prediction fitting the sequence of decision trees.

The estimation results using the above-mentioned methods can be found in Section 5 and Section 6. In the case of this study, observing the temperature curves of the stator winding and the temperature on the motor casing (Figure 4a,b), one can see an almost perfectly linear relationship between these variables. Therefore, the authors of this article decided to compare the effectiveness of machine learning algorithms in predicting the temperature of the stator winding of a BLDC motor, taking into account the temperature on the casing as well as in the complete absence of additional sensors. It is worth adding that by using the measurement data available from the converter system, Hall sensors, and transformations described in the previous section, the cost of the drive does not increase. This information is available in any drive system that uses current and speed regulators in the control algorithm.

## 4. Hyperparameters Optimization with Cross-Validation

Achieving the maximum possible predictive accuracy of each model is possible only through hyperparameter tuning, such as the regularization strength described in the previous section. These are parameters that influence the behavior of the algorithm and are not optimized in the learning process—their selection is the programmer’s task. For this purpose, it is necessary to carry out a series of tests to identify for which parameter values the temperature prediction error will be the smallest. However, it should be remembered that if during each of the trials, the effectiveness of the algorithms is validated on the same test set, the selected set of hyperparameters will be the best but only for this specific set and may turn out to be inappropriate for predicting new samples. Moreover, the test set has a limited size and will never be able to reflect all the dependencies that occurred in the training data. Therefore, it can be concluded that the model’s ability to generalize will be small when its hyperparameters are optimized for a specific case of the test set. One of the solutions to this problem may be to split it into three subsets: training, validation (used to optimize parameters), and test. However, the application of this method requires a significant number of samples, and in addition, the accuracy of the predictions may still depend on some random selection of the dataset split points. In practice, the most frequently used and extremely effective method to avoid problems with generalization is cross-validation.

**Figure 5:**
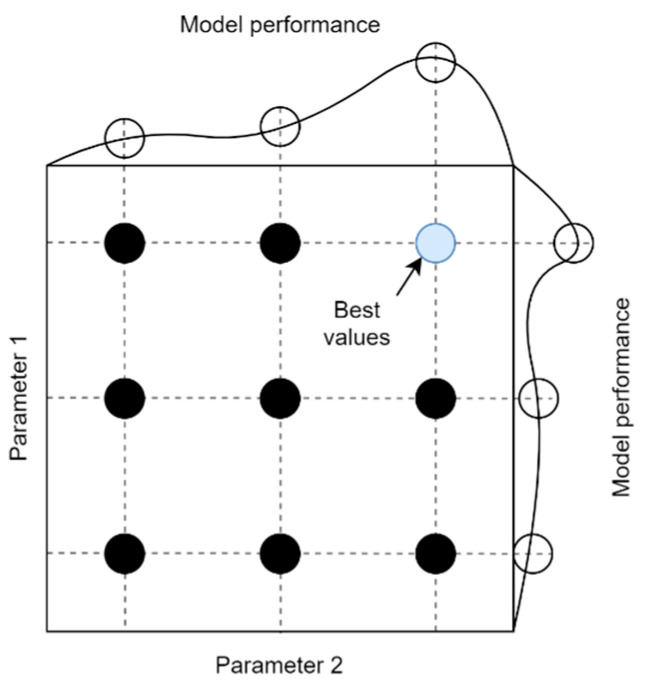
Example of a searching grid for two parameters.

**Figure 6:**
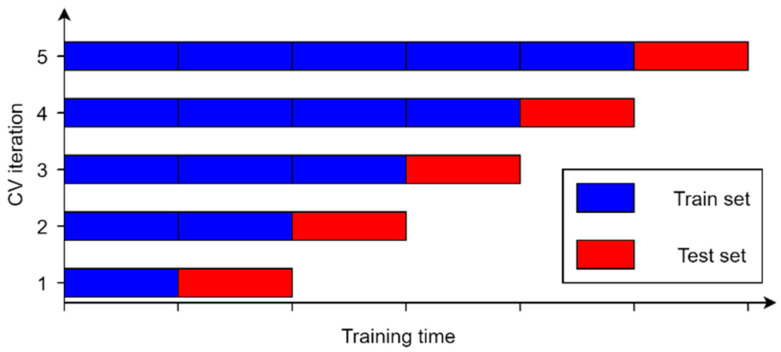
Time-series five-fold cross-validation.

In this study, an exhaustive gird search method with cross-validation was used to optimize the hyperparameters. For each of the algorithms, a grid consisting of different values of individual parameters was defined. An example of a grid for two parameters is shown in Figure 5. Of course, the dimensionality of the grid depends on the number of unique parameters that can be optimized and is different for each algorithm. In order to determine the effectiveness of the model for a given grid node, cross-validation with respect to the chronology of the data was used. This method consists in dividing the data into k groups in such a way that the algorithm is trained on a k subset and tested on k+1. The average of the performance metrics computed in each iteration determines the final score for that grid node. Thanks to this, unlike the traditional k-fold cross-validation, it is possible to prevent the algorithm from being tested on data older than training data. The principle of data division according to the described method for the applied value k=5 is shown in Figure 6. Based on the results for each node of the grid, a set of hyperparameter values is selected for which the compromise between the bias and variance of the model is the most optimal. Then, the algorithm is tested on a set of test data that has been set aside and which it has never actually seen before. This allows obtaining reliable results of the temperature prediction of the BLDC motor winding.

## 5. Results of the Sensorless Estimation Model

The evaluation of the efficiency of BLDC motor winding temperature estimation was carried out on the basis of the following regression metrics:

- RMSE (root mean squared error) defined as:

(7) $$\mathrm{RMSE}=\sqrt{\frac{1}{N}\sum_{i=1}^{N}{\left(\hat{y}-y\right)}_{i}^{2}}.$$

- MAPE (mean absolute percentage error) calculated with the formula:

(8) $$\mathrm{MAPE}=\left(\frac{1}{N}\sum_{i=1}^{N}{\left|\frac{\hat{y}-y}{y}\right|}_{i}\right)*100\%.$$

- Coefficient of determination R^2^ (quality of fit) calculated as:

(9) $$R^{2}=1-\frac{\sum_{i=1}^{N}{\left(\hat{y}-y\right)}_{i}^{2}}{\sum_{i=1}^{N}{\left(y-\bar{y}\right)}_{i}}.$$

In the above equations, N represents the number of samples, $\hat{y}$ is the predicted value, y is the actual value, and the

(10) $$\bar{y}=\frac{1}{N}\sum_{i=1}^{N}y_{i}$$

is the mean of the actual values.

**Figure 7:**
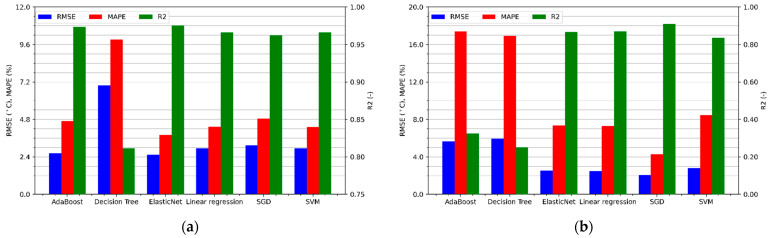
Regression metrics values for the sensorless temperature estimation of the motor: (**a**) without cooling; (**b**) with a cooling fan.

Figure 7a shows the performance metrics of winding temperature regression for a BLDC motor without additional cooling. As can be seen, all linear models are performing better than decision trees. Only the utilization of many trees and the use of boosting in the AdaBoost algorithm significantly improve the results, allowing for a similar effectiveness as linear models, each of which achieves the value of the determination coefficient above 0.96, the mean absolute percentage error below 5%, and the RMSE error not exceeding 3.2 °C.

Results of the temperature estimation of the motor cooled by the fan placed on the shaft are shown in Figure 7b. One can notice a decrease in the effectiveness of all predictive algorithms, in particular those that use decision trees. The R^2^ coefficient of linear models ranges from 0.84 to 0.91, while the MAPE error ranges from 4.3% to 8.5%. On the other hand, the root mean squared errors seem very interesting because they are smaller than in the case of temperature estimation without cooling. This means that the algorithm makes less error on average but has a much bigger problem with fitting, which also causes increased relative errors. The presented difference may result from the fact that the winding temperature of the motor cooled by a fan placed on the shaft depends to a greater extent on the rotational speed. Therefore, the relationship between the features and the target variable, sought by the algorithms, may be of a more complex nature, reducing the effectiveness of the estimation.

The highest accuracy of motor temperature prediction without cooling was achieved by the ElasticNet algorithm, for which the regression metrics are respectively RMSE = 2.53 °C, MAPE = 3.82%, and R^2^ = 0.975. Analyzing Figure 7b, it can be concluded that the accuracy of the motor temperature prediction with a cooling fan is the highest for the SGD algorithm. It achieved a coefficient of determination equal to 0.909, an RMSE error of 2.07ׄ °C, and a MAPE of 4.3%.

**Figure 8:**
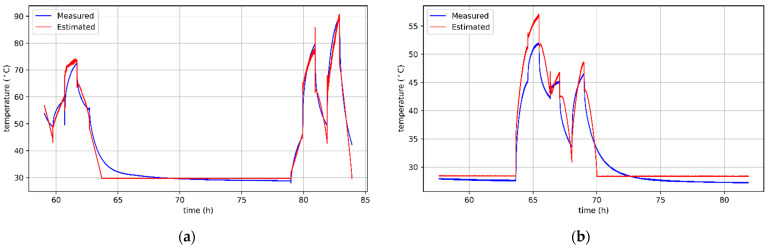
Results of sensorless temperature estimation for the motor: (**a**) without cooling, obtained with ElaticNet regressor; (**b**) with a cooling fan, obtained with SGD regressor.

Figure 8a,b show the curves of actual and predicted winding temperatures of a BLDC motor without cooling and with a cooling fan. It is worth noting that some errors are the result of incorrect temperature predictions of an unpowered motor during cooling to the ambient temperature. However, in practice, keeping track of the temperature within this range is not necessary in most applications. On the other hand, there are many important situations in which the motor operates at higher temperatures and should be protected from overheating. The algorithms’ behavior in this respect is beneficial, because they more often overestimate the predicted temperature. Thanks to this, the motor protection will be preserved because the information about too high temperature will appear earlier.

## 6. Results of the Estimation Model with Auxiliary Temperature Sensor

**Figure 9:**
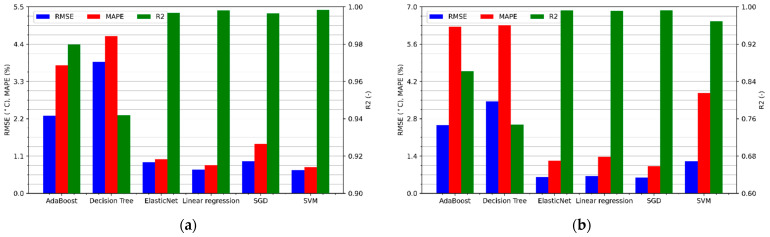
Regression metrics values for the temperature estimation supported with casing sensor data of the motor: (**a**) without cooling; (**b**) with a cooling fan.

The values of regression accuracy metrics for predicting the winding temperature of a BLDC motor without cooling and taking into account the temperature information on the casing are shown in Figure 9a and with cooling in Figure 9b.

**Figure 10:**
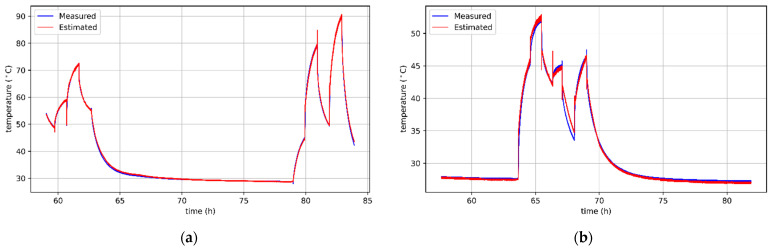
Results of temperature estimation supported with casing sensor data for the motor: (**a**) without cooling, obtained with SVM regressor; (**b**) with a cooling fan, obtained with SGD regressor.

As in the case of sensorless estimation, the results obtained using algorithms based on decision trees are much worse than linear models. In the third section, it was emphasized that on the basis of the winding and the motor casing temperature curves, an almost linear relationship between these variables can be noticed. The estimation results confirm these assumptions because the efficiency of linear models is very good. The RMSE value is less than 1.5 °C and the MAPE is less than 3.8%. Moreover, the coefficient of determination R^2^ for each linear algorithm is greater than or equal to 0.97. The best algorithm for estimating the motor temperature without cooling is undoubtedly the linear SVM. Its regression metrics are RMSE = 0.68 °C, MAPE = 0.77%, and R^2^ = 0.998, respectively. The curves of the actual and estimated winding temperatures by this algorithm are shown in Figure 10a. Thus, an almost perfect representation of the actual temperature is visible. On the other hand, a slight deterioration in efficiency is visible in all models estimating the motor temperature with an additional cooling fan. The best results during this test were obtained with the linear model optimized with the stochastic gradient descent algorithm, for which RMSE = 0.59 °C, MAPE = 1.02% and R^2^ = 0.993. The actual temperature and predicted temperature with the use of SGD are presented in Figure 10b. The decrease in the estimation accuracy for the cooled test is particularly interesting. As noted in the previous section, mounting a cooling fan on the shaft increases the effect of rotational speed on motor temperature and therefore increases the importance of this feature in prediction. Moreover, taking into account the temperature on the motor casing results in a significant improvement in the prediction accuracy. However, it should be remembered that the motor is cooled from the outside, so the rotational speed will have a greater effect on the temperature on the casing than on the inside of the motor. Therefore, it can be inferred that due to changes in rotational speed, the dependence of the winding temperature on the casing temperature will be more non-linear than in the case of a system without cooling. The above phenomenon may cause a significant estimation accuracy decrease.

## 7. Conclusions

This article proposes two models for BLDC motor winding temperature estimation using machine learning methods. The former allows the prediction of temperature without the need for temperature sensors, while the latter involves mounting an auxiliary sensor on the motor casing to improve prediction efficiency. In order to create an appropriate dataset for algorithm training, over 160 h of BLDC motor temperature measurements were carried out for a variable load and speed profile as well as various cooling conditions. Subsequently, data preprocessing was done as follows: additional features containing information about previous states of the device were introduced to create a short-term history of the device, the dataset was divided into training and test data, and then standardization was performed. Selected machine learning algorithms were used to estimate the temperature of the BLDC motor winding, namely: AdaBoost, decision tree, ElasticNet, linear regression, SGD, SVM. For each of the above-mentioned algorithms, a hyperparameter tuning process was performed through the use of a cross-validated grid search mechanism. As a result, it was possible to define a set of parameters that would ensure the appropriate generalization capability. 

For the sensorless temperature prediction of BLDC motor without cooling, the greatest effectiveness was achieved by the ElasticNet algorithm reaching MAPE = 3.82%, while the remaining linear models obtained similar, but slightly worse results. In the case of the test with an additional fan on the motor shaft, the SVM turned out to be the best algorithm, for which the mean absolute percentage error was 4.3%. It is worth noting that the obtained error values are comparable with the results of other methods described in the first section of this study. However, they allow completely avoiding the need to use temperature sensors and do not require influencing the current and voltage waveforms of the motor.

The authors of this study also decided to compare the effectiveness of the algorithms taking into account the information about the temperature on the motor casing. In this case, as suspected, the results turned out to be much better. The MAPE error of the best linear models did not exceed 1.5% for each case, while the RMSE was below 0.7 °C. It is worth noting that this good winding temperature prediction results can be used to compensate the temperature effect on the machine output torque.

Comparing the proposed models for motor winding temperature estimation to those described in the literature (Table 3), it can be concluded that the second model gives better [29] or similar [31] results to recurrent and convolutional neural networks. Moreover, this model performs a little worse than the deep neural networks used in [9] and similar to [30] (smaller mean absolute errors, but larger maximum error). However, it is worth mentioning that the algorithms used in model two are much less complex and need fewer resources for training and validation. In addition, model two provides comparable results to those obtained by the authors in [11,20,21] using thermal models of the motor. However, the superiority of the proposed model is that it does not require expertise knowledge of the modeled object. Both models also provide better results than the signal injection method presented in [23], and additionally, it does not require interference with the motor power system. The proposed sensorless model (model 1) gives similar results to the linear models presented in [34], but it does not require any additional temperature sensors if the ambient temperature does not significantly affect the motor temperature. However, it is important to note that the comparison of the results with those available in the literature is indicative, because different engine types and models were tested among the researchers.

Therefore, it has been proven that overheating protection of the motor can be provided using a trained machine learning algorithm without any additional sensors, thus avoiding the cost of installing additional hardware by the manufacturer or the user. In addition, it has been proven that the use of information from the sensor mounted on the BLDC motor casing allows for very good winding temperature prediction results. This means that using the second described model, it is possible to introduce a compensation mechanism for the temperature impact on the motor output torque. It is worth adding that mounting the sensor on the motor casing is an uncomplicated operation that the user who wants to know the exact temperature inside the device can do by himself and at a low cost. In addition, most motor faults, such as interturn short circuits, bearing damage or magnet degradation, cause the motor temperature to rise significantly. Therefore, it can be anticipated that the described temperature estimation method can be used to detect device components damages.

The increase in ambient temperature may be problematic for the described estimation method. Under laboratory conditions, the ambient temperature was approximately constant and had no effect on the casing temperature and inside the motor. Difficult conditions at the motor site may increase the prediction errors, but a possible solution is to introduce an additional variable informing about the ambient temperature. In addition, future research should verify the effectiveness of temperature estimation for a fast-varying load profile and more sophisticated methods such as neural networks.

## Figures and Tables

**Table 1:** Tested BLDC motor specifications.

Name	Unit	Value
No. of pole	-	8
No. of phase	-	3
Rated voltage	V	48
Rated speed	rpm	3000
Rated torque	Nm	1.4
Max peak torque	Nm	4.2
Torque constant	Nm/A	0.127
Line to line resistance	Ω	0.16
Line to line inductance	mH	0.50
Max peak current	A	33
No-load current	mA	1450
Length	Mm	98
Rotor inertia	g cm^2^	1600
Weight	Kg	3.15

**Table 2:** Regularized objective functions.

Regularization	Linear Regressor	Objective Function
L2	Ridge	$\min{\left\|\hat{y}-y\right\|}_{2}^{2}+\alpha {\left\|w\right\|}_{2}^{2}$
L1	Lasso	$\min\frac{1}{2N}{\left\|\hat{y}-y\right\|}_{2}^{2}+\alpha {\left\|w\right\|}_{1}$
L1 + L2	ElasticNet	$\min\frac{1}{2N}{\left\|\hat{y}-y\right\|}_{2}^{2}+\alpha \rho {\left\|w\right\|}_{1}+\frac{\alpha (1-\rho )}{2}{\left\|w\right\|}_{2}^{2}$

**Table 3:** Comparison of estimation results with methods reported in the literature.

Method		Metric						
		MSE ^a^	RMSE	MAPE	R^2^	$\underset{i}{\max}{\left|\hat{y}-y\right|}_{i}$	MAE ^b^	MRE ^c^
Literature method	[9]	-	0.24 °C	-	0.944	-	0.15 °C	-
	[11]	-	-	-	-	5.2 °C	-	1.50%
	[20]	-	-	-	-	≈8.0 °C	-	-
	[21]	-	-	-	-	8.0 °C	-	-
	[23]	-	-	-	-	-	-	6.14%
	[29]	2.04 K^2^	1.43 K	-	-	37.6 K	-	-
	[30]	-	-	-	-	4.5 °C	0.90 °C	-
	[31]	-	-	-	-	10.8 K	-	-
	[34]	6.06 K^2^	2.46 K	-	-	11.1 K	-	-
Model 1	Uncooled (ElasticNet)	6.40 °C^2^	2.53 °C	3.82%	0.975	20.4 °C	1.64 °C	3.82%
	Cooled (SGD)	4.28 °C^2^	2.07 °C	4.30%	0.909	8.3 °C	1.49 °C	4.30%
Model 2	Uncooled (SVM)	0.46 °C^2^	0.68 °C	0.77%	0.998	14.0 °C	0.34 °C	0.77%
	Cooled (SGD)	0.35 °C^2^	0.59 °C	1.02%	0.993	7.3 °C	0.35 °C	1.02%

^a^ mean squared error, ^b^ mean absolute error, ^c^ mean relative error.

## Data Availability

The database developed for this article [42] is available from IEEE Dataport at https://ieee-dataport.org/documents/bldc-motor-temperature (accessed on 6 July 2021).
